# Incidence and Radiological Risk Factors of Proximal Junctional Kyphosis in Adolescent Idiopathic Scoliosis Following Pedicle Screw Instrumentation with Rod Derotation and Direct Vertebral Rotation: A Minimum 5-Year Follow-Up Study

**DOI:** 10.3390/jcm10225351

**Published:** 2021-11-17

**Authors:** Hong Jin Kim, Jae Hyuk Yang, Dong-Gune Chang, Se-Il Suk, Seung Woo Suh, Ji Su Kim, Sang-Il Kim, Kwang-Sup Song, Woojin Cho

**Affiliations:** 1 Department of Orthopedic Surgery, College of Medicine, Inje University Sanggye Paik Hospital, Inje University, Seoul 01757, Korea; hongjin0925@naver.com (H.J.K.); seilsuk@unitel.co.kr (S.-I.S.); jsk4417@naver.com (J.S.K.); 2 Department of Orthopedic Surgery, College of Medicine, Korea University Guro Hospital, Korea University, Seoul 08308, Korea; kuspine@naver.com (J.H.Y.); spine@korea.ac.kr (S.W.S.); 3 Department of Orthopedic Surgery, College of Medicine, The Catholic University of Korea, Seoul 06591, Korea; sang1kim81@gmail.com; 4 Department of Orthopedic Surgery, College of Medicine, Chung-Ang University Hospital, Chung-Ang University, Seoul 06973, Korea; ksong70@cau.ac.kr; 5 Montefiore Medical Center, Department of Orthopedic Surgery, Albert Einstein College of Medicine, Bronx, NY 10467, USA; woojinchomd@aol.com

**Keywords:** adolescent idiopathic scoliosis, pedicle screw instrumentation, rod derotation, direct vertebral rotation, proximal junctional kyphosis

## Abstract

Several studies have reported incidence and risk factors for the development of proximal junctional kyphosis (PJK) in patients with adolescent idiopathic scoliosis (AIS). However, there is little information regarding long-term follow-up after pedicle screw instrumentation (PSI) with rod derotation (RD) and direct vertebral rotation (DVR). Sixty-nine AIS patients who underwent deformity correction using PSI with RD and DVR were retrospectively analyzed in two groups according to the occurrence of PJK, with a minimum five-year follow-up, including a non-PJK group (*n* = 62) and PJK group (*n* = 7). Radiological parameters were evaluated at preoperative, postoperative, and last follow-up. Incidence for PJK was 10.1% (7/69 patients), with a mean 9.4-year follow-up period. The thoracolumbar/lumbar curve (TL/L curve) was proportionally higher in the PJK group. The proximal compensatory curve was significantly lower in the PJK group than in the non-PJK group preoperatively (*p* = 0.027), postoperatively (*p* = 0.001), and at last follow-up (*p* = 0.041). The development of PJK was associated with the TL/L curve pattern, lower preoperative proximal compensatory curve, and over-correction of the proximal curve for PSI with RD and DVR. Therefore, careful evaluation of compensatory curves as well as of the main curve is important to prevent the development of PJK in the treatment of AIS.

## 1. Introduction

Proximal junctional kyphosis (PJK) has been considered a complication that reflects pathological changes which develop around the adjacent segment after long instrumented posterior fusion [1,2]. The incidence of PJK has varied from 1.8% to 46% in adult populations because the criteria of PJK accepted according to the proximal junctional angle (PJA) have not been universally accepted [2,3,4]. The development of PJK has also been affected by several factors such as patient-specific, radiological, and surgical factors [2].

Pedicle screw instrumentation (PSI) was the mainstay of surgical treatments in patients with adolescent idiopathic scoliosis (AIS). PSI showed better outcomes by three-dimensional mechanical fixation with shorter fusion and less blood loss compared to traditional techniques such as Harrington instrumentation, Cotrel–Dubosset instrumentation, and hook/wire fixation [5,6,7,8]. Rod derotation (RD) and direct vertebral rotation (DVR) made direct correction of spinal axial rotation possible, which provided a more accurate correction of rotational deformity and sagittal alignment, improved clinical outcomes, and decreased the rib humps [7,8]. RD and DVR after PSI also enables reduced fusion levels while minimizing the loss of correction as well as complications during growth [5,6,7,8]. However, PSI has serious pedicle screw-related complications including malposition, neural injury, and dislodged or prominent instrumentation [9]. Furthermore, pedicle screw constructs lead to a higher incidence rate of PJK compared to other instrumentation systems [4].

Several studies have reported the incidence and risk factors for the development of PJK in patients with AIS following deformity correction [3,10,11,12,13,14,15,16]. However, few reports have focused on the development of PJK during the long-term follow-up of patients with AIS following PSI with RD and DVR. Therefore, this study aimed to investigate incidence and radiological factors of PJK in AIS following PSI with RD and DVR through a minimum 5-year follow-up period.

## 2. Materials and Methods

**Figure 1:**
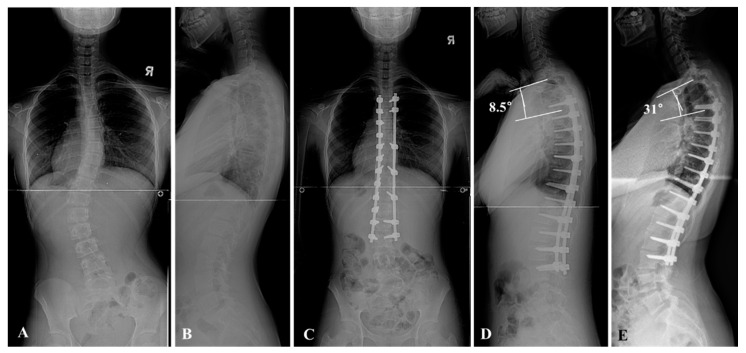
A 13-year-old female patient presented to the orthopedic clinic due to incidental findings of adolescent idiopathic scoliosis (AIS). The whole spine anteroposterior and lateral views showed the thoracolumbar and lumbar curve (TL/L curve) pattern. The Cobb’s angle was 42° (**A**,**B**). Posterior segmental instrumentation (PSI) with direct vertebral rotation (DVR) and rod derotation (RD) from T4 to L3 was performed, and the curvature was corrected to 3° after surgery (**C**). The proximal junctional angle from T2 to T4 was 8.5° in the postoperative whole spine lateral view (**D**). On 5-year follow-up, the proximal junctional angle from T2 to T4 was progressed to 31° in the follow-up whole spine lateral view (**E**).

**Figure 2:**
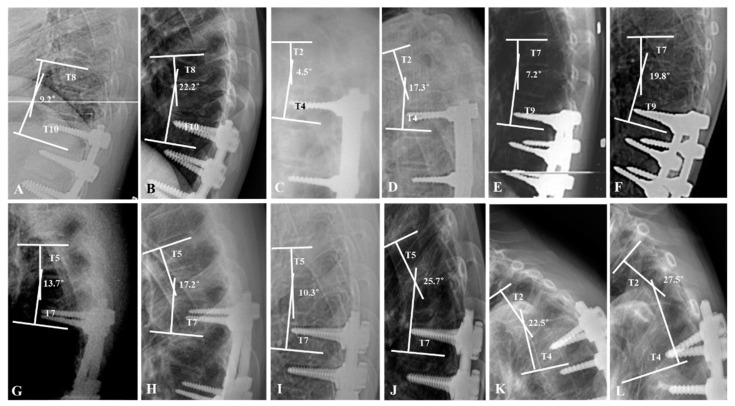
The profiles of the proximal junctional kyphosis (PJK) groups. A 15-year-old female patient underwent deformity correction from T10 to L4. The proximal junctional angle (PJA) was progressed from 9.2° to 22.2° at 1-year follow-up (**A**,**B**). A 14-year-old female patient underwent deformity correction from T4 to L3. PJA was progressed from 4.5° to 17.3° at 2-year follow-up (**C**,**D**). A 13-year-old female patient underwent deformity correction from T9 to L4. PJA was progressed from 7.2° to 19.8° at 6-month follow-up (**E**,**F**). A 15-year-old male patient underwent deformity correction from T7 to L2. PJA was progressed from 13.7° to 17.2° at 6.5-year follow-up (**G**,**H**). A 13-year-old female patient underwent deformity correction from T7 to L3. PJA was progressed from 10.3° to 25.7° at 1-year follow-up (**I**,**J**). A 14-year-old female patient underwent deformity correction from T4 to T12. PJA was progressed from 22.5° to 27.5° at 6-month follow-up (**K**,**L**).

This study was performed through a retrospective comparative analysis at a single institute where spinal deformity corrections are routinely performed. All deformity correction procedures were performed by a senior spine surgeon with vast experience in performing standard open surgeries. The concept and procedures of the study were approved by our Institutional Review Board (2018-10-013). Medical record data of 69 patients with AIS who underwent deformity correction using bilateral PSI with RD and DVR from 2002 to 2012 were retrospectively analyzed. The patients were divided into non-PJK (*n* = 62) and PJK groups (*n* = 7) according to the occurrence of PJK during a minimum five-year follow-up period. In our study, PJA was defined as the Cobb’s angle between the lower end plate of the uppermost instrumented vertebrae (UIV) and upper end plate of the above two vertebrae of UIV. The criteria for PJK were a postoperative PJA of more than 10 degrees than the preoperative PJA or an absolute value of PJA greater than 10 degrees, as proposed by Glattes et al. [1,2,3] (Figure 1 and Figure 2).

All patients underwent PSI with RD and DVR by the posterior approach. Fusion levels were determined according to the Suk classification. Pedicle screws (reduction mono-axial screws) were inserted segmentally on both sides of the lumbar curve and on the concave side, as well as in every other or every third vertebra on the convex side in the thoracic curve. A contoured rod (titanium-alloy rods) to one-third more than the normal sagittal alignment was inserted into the correction side (concave side in the thoracic curve and convex side in the lumbar curve) and derotated 90° to transform the scoliotic curve into thoracic kyphosis and/or lumbar lordosis. After correcting the coronal and sagittal curves by RD, DVR was implemented to correct rotational deformity. The direction of DVR was opposite to the rotation of the vertebrae in the transverse plane. The derotator was pulled towards the desired direction for rotational correction of the vertebral body after attaching the derotator to the screw head on the correction side. The rod was then bent to conform to the shape of the corrected curve e and placed in situ without forceful manipulation in the supportive side [17]. All patients wore a thoracolumbosacral orthosis brace for three months after surgery without any specific rehabilitation.

All of the patient data were collected from the hospital database and retrospectively analyzed in 2020. Demographics and operative data included age, follow-up period, Risser stages, operative time, estimated blood loss, thoracoplasty, and number of resected ribs. Radiographic variables included coronal, sagittal, and balance parameters at preoperative, postoperative, and last follow-up. The main thoracic curve, proximal curve, and distal compensatory curves (PCC and DCC) were collected as coronal parameters. The main thoracic curve was assessed by the Cobb’s angles of the thoracic curve. The UIV tilt angle, UIV disc angle, lowest instrumented vertebra (LIV) tilt angle, and LIV disc angle were also assessed. Data on PJA and the presence of thoracic kyphosis (TK) and lumbar lordosis (LL) were collected as sagittal parameters. Data on the coronal balance (CB) and sagittal vertical axis (SVA) were collected as balance parameters.

Statistical analysis was performed using SPSS Statistics for Windows, version 21.0 (IBM Corp., Armonk, NY, USA). Normal distribution was confirmed by the Kolmogorov–Smirnov test. Regarding continuous variables, the Student’s *t*-test and Mann–Whitney test were used for parametric data and non-parametric data when appropriate. Regarding categorical variables, the chi-square test and Fisher’s exact test were used for parametric and non-parametric data when appropriate. In the case of variables having negative or positive values based on the measured reference point, such as coronal balance and SVA, statistical comparisons of groups required converting negative numbers to positive numbers because of the necessity to statistically analyze differences from a reference point. Statistical significance was set at *p* < 0.05.

## 3. Results

**Figure 3:**
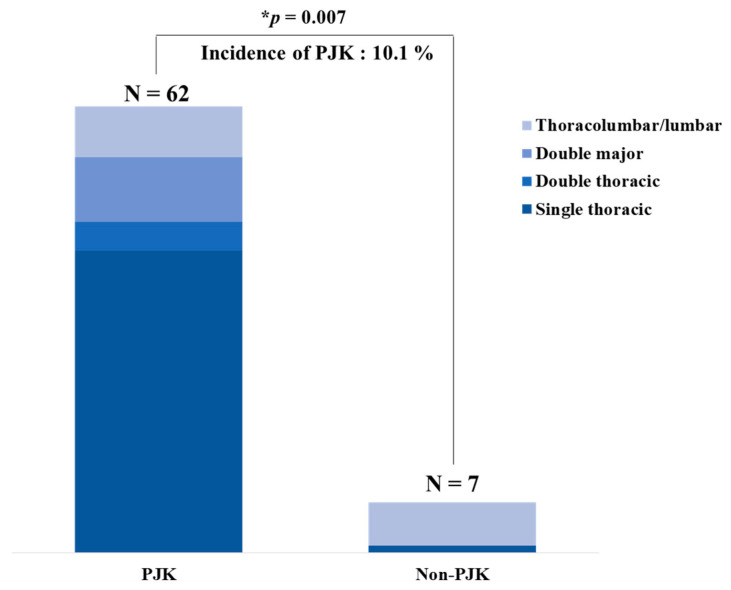
Incidence rate of proximal junctional kyphosis (PJK) in the patients with adolescent idiopathic scoliosis (AIS) following posterior segmental instrumentation (PSI) with direct vertebral rotation (DVR) and rod derotation (RD). Seven of 69 patients showed development of PJK (10.1%) within a mean 9.4-year follow-up period, showing a proportionally higher thoracolumbar/lumbar curve with statistical significance (* *p* = 0.007). N = number; * means *p* value < 0.05.

Seven of 69 patients had a PJK (10.1%) within the 9.4-year mean follow-up period, showing a proportionally higher thoracolumbar/lumbar curve (Figure 3).

In Suk’s classification, the thoracolumbar/lumbar curve was distributed in 11.3% in the non-PJK and 85.7% in the PJK group. There was a statistical difference between non-PJK and PJK groups in Suk’s classification (*p* = 0.007). The mean age in the non-PJK and PJK group was 14.2 years and 13.9 years, respectively (*p* = 0.890). The follow-up duration for the non-PJK and PJK groups was 9.4 years and 8.3 years, respectively (*p* = 0.329). The Risser stage in the non-PJK and PJK groups was 2.6 and 2.1, respectively (*p* = 0.503). Regarding the operative data, there were 11.4 and 11.5 fusion segments in the non-PJK and PJK groups, respectively, with statistical significance (*p* = 0.02). There were no significant differences in the operative time (*p* = 0.116) and estimated blood loss (*p* = 0.078) between the two groups. Thoracoplasty was performed in 72% of total patients with a mean of 6.2 resected ribs. There were no significant differences in the thoracoplasty (*p* = 0.085) and number of resected ribs between the two groups (*p* = 0.747; Table 1).

Regarding the coronal parameters, there were no significant differences in the preoperative, postoperative, and last follow-up main thoracic curve (all *p* > 0.05). Flexibility of the main thoracic curve in the non-PJK and PJK group was 37.2% and 40.3%, respectively (*p* = 0.522). The preoperative PCC was significantly lower in the PJK group (17.7°) than in the non-PJK group (26°; *p* = 0.027). The postoperative PCC was also significantly lower in the PJK group (12.4°) than in the non-PJK group (4°; *p* = 0.001). The last follow-up PCC was also significantly lower in the PJK group (16.6°) than in the non-PJK group (7°; *p* = 0.041). There were no significant differences in the correction angle (*p* = 0.979) and loss of correction in PCC (*p* = 0.688; Table 2).

There were no significant differences among all the parameters regarding the tilt and disc angles of UIV and LIV between the non-PJK group and PJK group (all *p* > 0.05; Table 3).

Regarding the balance parameters, preoperative CB was 12.6 mm in the non-PJK and 19.1 mm in the PJK group (*p* = 0.166). Postoperative CB showed within normal limits in both groups (10.7 mm in the non-PJK and 6.0 mm in the PJK group; *p* = 0.072). Preoperative SVA was −2.6 mm in the non-PJK and −20.4 mm in the PJK group (*p* = 0.127). Postoperative SVA showed within normal limit in both groups (5.1 mm in the non-PJK and 4.2 mm in the PJK group; *p* = 0.767). Regarding the sagittal parameters, preoperative PJA was higher in the PJK group (7.3°) than in the non-PJK group (4.4°) with no significance (*p* = 0.063). However, postoperative PJA was significantly higher in the PJK group (11.4°) than in the non-PJK group (4.8°; *p* = 0.01). Last follow-up PJA was 19.1° in the PJK group and 4.7° in the non-PJK group with statistical significance (*p* < 0.001). There were no significant differences in TK, LL, and pelvic tilt (all *p* > 0.05); Table 4).

## 4. Discussion

Although PJK has been identified in patients with AIS following deformity correction, there has been debate on the risk factors for the development of PJK in AIS [1,2]. Some of the possible causes of PJK in AIS were reported to be scoliosis type, surgical technique, preoperative hyperkyphosis (≥40°), reduction of thoracic kyphosis after surgery, pedicle screw construct, thoracoplasty, and level of fused segments (≥12) [12,13,14,15,16]. However, the incidence and risk factors of PJK in the AIS population with long-term follow-up remain unclear [17]. This study aimed to elucidate incidence and risk factors of PJK following PSI with RD and DVR through long-term follow-up duration. In our study, we retrospectively analyzed the development of PJK and the associated risk factors in patients with AIS following PSI with RD and DVR over the course of an average of 9.4 years.

Our study showed that the incidence rate of PJK was 10.1% during a 9.4-year follow-up period in patients with AIS following PSI with RD and DVR. This result was comparable with the 13.1% of the Alzakri A et al. case control study in which the surgical technique was performed by PSI [16]. However, the reported incidence of PJK has varied from 7.1% to 46% [2]. The causes for the variations in the incidence rate may be multi-factorial including scoliosis type, surgical technique, and radiological imbalances [1,4,17]. Higher incidences of PJK may possibly occur due to the broader criteria of Cobb’s angle as there has been no universal definition in PJK [10,14]. Lee et al. set the criterion for abnormal kyphosis as more than 5°, which showed a 46% of incidence rate [10]. Lonner et al. showed a relatively low incidence (7.1%) of PJK, which depended on the surgeon’s operative technique including the selection of fusion level, rod contour, preservation of posterior elements, and use of screws or hook anchors [14]. DVR, as a new technique of three-dimensional deformity correction with PSI in AIS, was developed by Suk. et al., providing better rotational and coronal correction than PSI with RD [17,18]. However, the incidence rate of PJK has been reported similarly. Therefore, DVR did not have a substantial effect on PJK because the level of UIV was restricted by the rib.

In our retrospective long-term follow-up study, the onset of PJK was observed in two patients within 6 months postoperatively; two within one year postoperatively; two within 3 years postoperatively; and one in 6 years postoperatively. PJK usually occurs within 2 years postoperative but 42.9% of patients showed PJK after 2 years postoperative in AIS [2]. All patients in the PJK group were asymptomatic, thus we observed the progress and examined the plain radiographs every year in the outpatient clinic. Therefore, AIS patients following PSI with RD and DVR need long-term follow-ups to diagnose the progression to PJK.

There have been some reports of a substantial effect of curve patterns on PJK in AIS patients after surgery [14]. In our study, the thoracolumbar and lumbar curve in Suk’s classification had a relatively high proportion of PJK. The thoracolumbar and lumbar curve was also associated with lower PCC curves. This was consistent with preoperative PCC results. To the best of our knowledge, the incidence of PJK after AIS surgery according to Suk’s classification has rarely been well reported. The thoracolumbar and lumbar curve also matched with only Lenke’s classification type 5; this may be related to fusion extension in the proximal to natural thoracic kyphosis apex [7,19,20]. Furthermore, Zhao et al. showed a higher rate of PJK in Lenke’s 5 classification after PSI, which has a relatively higher incidence of PJK (40.2%) [20]. Therefore, our results showed that thoracolumbar and lumbar curves had a relatively higher incidence rate of PJK in accordance with Lenke’s classification type 5 and a preoperative lower PCC curve. Furthermore, the PJK group showed overcorrection despite the preoperative lower PCC curve, which means that proper correction is important to prevent PJK.

Thoracoplasty is mainly affected on the correction of main thoracic curves because this technique is performed in the apical vertebrae of the main thoracic curve. Therefore, thoracoplasty is not associated with the correction of the PCC. Some reports have suggested that thoracoplasty destabilizes in the spine–chest cage and provides more stress in the instrumentation, leading to the development of PJK. However, the effect of thoracoplasty is controversial due to its being performed within the level of fused segments, which suggests that thoracoplasty does not have a substantial effect on the development of PJK [2,19]. Kim et al. suggested an effect of thoracoplasty in AIS patients; significant development of PJK occurred with thoracoplasty and hybrid instrumentation (i.e., proximal hook and distal pedicle screws) [11,19]. However, from our results, thoracoplasty does not have an effect on the development of PJK in patients with AIS following PSI with RD and DVR. Therefore, thoracoplasty was not considered to be a main factor in the development of PJK in AIS compared to other factors such as preoperative hyperkyphosis, scoliosis type, and correction rate [20].

For the radiological parameters, preoperative hyper-thoracic kyphosis and the substantial correction of the thoracic kyphosis were reported as factors for the development of PJK [19]. In our data, preoperative thoracic kyphosis and the correction angle were higher in the PJK group than in the non-PJK group. However, there were no statistical differences for these parameters between the two groups. The DVR system allows for more a sophisticated correction of the sagittal alignment with PSI in AIS patients compared to other indirect techniques for the correction of spinal axial rotation [21,22,23,24]. From our results, preoperative thoracic kyphosis and the degree of correction were still important factors for the development of PJK, but PSI with RD and DVR was likely to reduce possible complications with sagittal alignment. However, future trials may need to evaluate the relationship between the effect of the DVR system and PJK.

This study had some limitations. First, our analysis was based on small sample-sized retrospective data. However, this study showed similar incidences within ranges of previously reported data for the incidence of PJK. Second, the measurement of the vertebra has an ambiguity that plain radiographs inherently possess [25,26]. Future work using three-dimensional images may also be needed. Third, the clinical outcomes were not assessed in this study. The main purpose of this study was to evaluate the prevalence and radiological risk factors of PJK related to PSI with RD and DVR. Clinical outcomes were not included. Further studies are needed to evaluate the correlations of clinical outcomes according to the measurements of PJA to elucidate the importance of PJK in AIS patients.

## 5. Conclusions

The development of PJK was associated with the TL/L curve pattern, lower preoperative proximal compensatory curve, and over-correction of the proximal curve for PSI with RD and DVR. Therefore, careful evaluation of compensatory curves as well as of the main curve is important to prevent the development of PJK in the treatment of AIS.

## Figures and Tables

**Table 1:** Demographic and operative data of comparison between the non-PJK and PJK group.

Variable	Non-PJK (*N* = 62)	PJK (*N* = 7)	*p* Value
Age (years)	14.2 ± 2.2	13.9 ± 0.9	0.890
Follow-up (years)	9.4 ± 2.8	8.3 ± 3.5	0.329
Risser stage	2.6 ± 1.5	2.1 ± 1.8	0.503
Suk classification Single thoracic Double thoracic Double major Thoracolumbar/lumbar	42 4 9 7	1 0 0 6	0.007 †
Lenke classifciation Type I Type II Type IIIType IV Type V Type VI	41 2 1 4 6 8	2 0 0 0 5 0	0.015 †
Fusion segments	11.4 ± 1.9	11.5 ± 2.4	0.020
Operative time (min)	222.1 ± 69.6	188.6 ± 50.1	0.116
Estimated blood loss (mL)	2093.0 ± 1314.8	1400.0 ± 838.6	0.078
Thoracoplasty (number of patients)	46	3	0.085
Number of resected ribs	6.2 ± 1.0	6.0 ± 1.0	0.747

† Data all represent mean values for each group. † *p* value is calculated by Fisher’s exact test. Significant differences are accepted for *p*-values < 0.05. PJK = proximal junctional kyphosis.

**Table 2:** Comparison of main thoracic, proximal, and lumbar curves between the non-PJK and PJK group.

Variable	Non-PJK (*N* = 62)	PJK (*N* = 7)	*p* Value
**Main thoracic curve (°)**			
Preoperative	50.2 ± 13.6	47.4 ± 13.0	0.129
Postoperative	11.9 ± 6.9	9.2 ± 9.1	0.169
Correction angle	38.4 ± 13.7	38.2 ± 7.2	0.688
Correction rate (%)	76.8 ± 11.6	82.8 ± 13.5	0.202
Last follow-up	14.1 ± 8.7	14.0 ± 10.0	0.973
Loss of correction	2.2 ± 5.9	4.8 ± 7.2	0.587
**Flexibility (%)**	37.2 ± 19.1	40.3 ± 20.6	0.522
**Proximal CC (°)**			
Preoperative	26.0 ± 10.8	17.7 ± 5.7	0.027
Postoperative	12.4 ± 6.4	4.0 ± 2.6	0.001
Correction angle	13.6 ± 10.5	13.7 ± 5.9	0.979
Correction rate (%)	53.6 ± 23.5	75.7 ± 15.3	0.018
Last follow-up	16.6 ± 26.7	7.0 ± 3.7	0.041
Loss of correction	4.4 ± 25.8	1.7 ± 5.2	0.688
**Distal CC (°)**			
Preoperative	28.5 ± 11.2	13.2 ± 10.7	0.006
Postoperative	7.8 ± 6.0	8.2 ± 8.1	0.926
Correction angle	20.7 ± 10.3	10.7 ± 14.9	0.007
Correction rate (%)	75.8 ± 16.7	54.8 ± 28.8	0.008
Last follow-up	9.3 ± 7.8	8.4 ± 8.9	0.926
Loss of correction	1.3 ± 7.6	1.3 ± 6.9	0.735

Significant differences are accepted for *p*-values < 0.05. CC = compensatory curve.

**Table 3:** Comparison of the tilt and disc angle of UIV and LIV between the non-PJK and PJK group.

Variable	Non-PJK (*N* = 62)	PJK (*N* = 7)	*p* Value
**UIV tilt (°)**			
Preoperative	14.8 ± 8.5	14.5 ± 11.2	0.643
Postoperative	5.9 ± 4.0 * (60.1%)	5.0 ± 3.2 * (65.5%)	0.55
Correction angle	8.9 ± 8.4 (39.9%)	9.5 ± 8.7 (34.5%)	0.901
Last follow-up	6.5 ± 4.3 (56.1%)	5.3 ± 4.7 (63.4%)	0.392
Loss of correction	0.6 ± 3.6	0.3 ± 4.1	0.443
**UIV disc angle (°)**			
Preoperative	3.7 ± 3.0	3.3 ± 3.0	0.742
Postoperative	3.2 ± 2.6 (13.5%)	2.1 ± 1.5 (26.4%)	0.293
Correction angle	0.5 ± 3.2 (86.5%)	1.2 ± 3.3 (63.6%)	0.817
Last follow-up	3.9 ± 3.0 (−5.4%)	3.2 ± 2.1 (3.0%)	0.605
Loss of correction	0.7 ± 2.5	1.1 ± 2.6	0.51
**LIV tilt (°)**			
Preoperative	18.2 ± 7.9	17.1 ± 10.1	0.915
Postoperative	4.3 ± 4.0 * (76.4%)	6.4 ± 4.3 (62.6%)	0.154
Correction angle	13.9 ± 7.2 (23.6%)	10.7 ± 10.2 (37.4%)	0.359
Last follow-up	5.4 ± 4.2 (70.3%)	6.7 ± 4.8 (60.8%)	0.428
Loss of correction	1.2 ± 4.2	0.3 ± 3.1	0.438
**LIV disc angle (°)**			
Preoperative	6.2 ± 4.9	4.6 ± 2.4	0.423
Postoperative	3.0 ± 2.8 * (51.6%)	3.2 ± 2.7 (30.4%)	0.769
Correction angle	3.2 ± 6.0 (48.4%)	1.5 ± 2.5 (69.6%)	0.137
Last follow-up	3.6 ± 3.1 (41.9%)	3.1 ± 1.7 (32.6%)	0.831
Loss of correction	0.6 ± 3.4	−0.1 ± 3.1	0.544

* Significantly changed from the value of the previous time point. Significant differences are defined as those with a *p*-value < 0.05. *N* = number; UIV = uppermost instrumented vertebra; and LIV = lowest instrumented vertebra.

**Table 4:** Comparison of the balance and sagittal parameters between the non-PJK and PJK group.

Variable	Non-PJK (*N* = 62)	PJK (*N* = 7)	*p* Value
**Coronal balance (mm)**			
Preoperative	12.6 ± 8.5	19.1 ± 10.9	0.166
Postoperative	10.7 ± 7.5	6.0 ± 5.8	0.072
Correction angle	2.5 ± 9.7	7.1 ± 19.3	0.199
Last follow-up	7.0 ± 5.5	8.9 ± 3.6	0.169
Loss of correction	−3.3 ± 8.6	−0.4 ± 14.6	0.123
**Sagittal vertical axis (mm)**			
Preoperative	−3.5 ± 28.4	−20.5 ± 24.1	0.127
Postoperative	5.3 ± 27.3	4.2 ± 38.7	0.767
Correction angle	8.9 ± 41.2	24.7 ± 54.8	0.367
Last follow-up	5.8 ± 22.5	−3.8 ± 33.6	0.717
Loss of correction	0.5 ± 37.5	−8.0 ± 62.3	0.345
**Thoracic kyphosis (°)**			
Preoperative	16.6 ± 10.4	27.2 ± 20.4	0.132
Postoperative	21.4 ± 8.1 *	25.3 ± 8.4	0.225
Correction angle	−4.9 ± 10.1	0.9 ± 15.0	0.407
Last follow-up	29.6 ± 32.6 *	28.2 ± 11.3	0.415
Loss of correction	8.0 ± 33.3	3.5 ± 5.8	0.908
**Lumbar lordosis (°)**			
Preoperative	47.2 ± 12.0	55.3 ± 14.2	0.166
Postoperative	46.2 ± 12.7	49.6 ± 12.0	0.563
Correction angle	1.1 ± 11.9	3.7 ± 13.9	0.748
Last follow-up	55.0 ± 11.7	58.9 ± 15.2	0.555
Loss of correction	9.1 ± 11.6	7.1 ± 14.1	0.515
**Pelvic tilt (°)**			
Preoperative	15.8 ± 2.6	13.5 ± 6.0	0.373
Postoperative	18.1 ± 5.4	17.1 ± 8.0	0.887
Last follow-up	14.5 ± 5.2	16.8 ± 7.9	0.522

* Significantly changed from the value of the previous time point. Significant differences are defined as those with a *p* value < 0.05.

## Data Availability

Data collected for this study, including individual patient data, will not be made available.
